# Supplementary material for: Design, Synthesis, Antibacterial, and Antitumor Activity of Linear Polyisocyanide Quaternary Ammonium Salts with Different Structures and Chain Lengths
Source: Molecules. 2021 Sep 19;26(18):5686. doi: 10.3390/molecules26185686 (PMC8472106; doi:10.3390/molecules26185686)
Supplement: Supplementary file 1 [file molecules-26-05686-s001.zip › molecules-1365386-supplementary.pdf]

molecules

Supplementary Materials: Design, Synthesis, Antibacterial, and Antitumor Activity of Linear Polyisocyanide Quaternary Ammonium Salts with Different Structures and Chain Lengths

Hongguang Zhang [1], Lijia Liu [2], Peng Hou [1], Jun Liu [1] and Shuang Fu [1,*]

[1] College of Pharmacy, Qiqihar Medical University, Qiqihar 161006, China; zhanghg@qmu.edu.cn (H.Z.); houp@qmu.edu.cn (P.H.); L_j2016@qmu.edu.cn (J.L.)

[2] Key Laboratory of Superlight Materials & Surface Technology, Ministry of Education, Institute of Advanced Marine Materials, College of Materials Science and Chemical Engineering, Harbin Engineering University, Harbin 150001, China; liulijia@hrbeu.edu.cn

* Correspondence: fsjt1980@qmu.edu.cn; Tel.: +86-45-2266-3152

(a) ^{1}H NMR spectra of the palladium catalyst

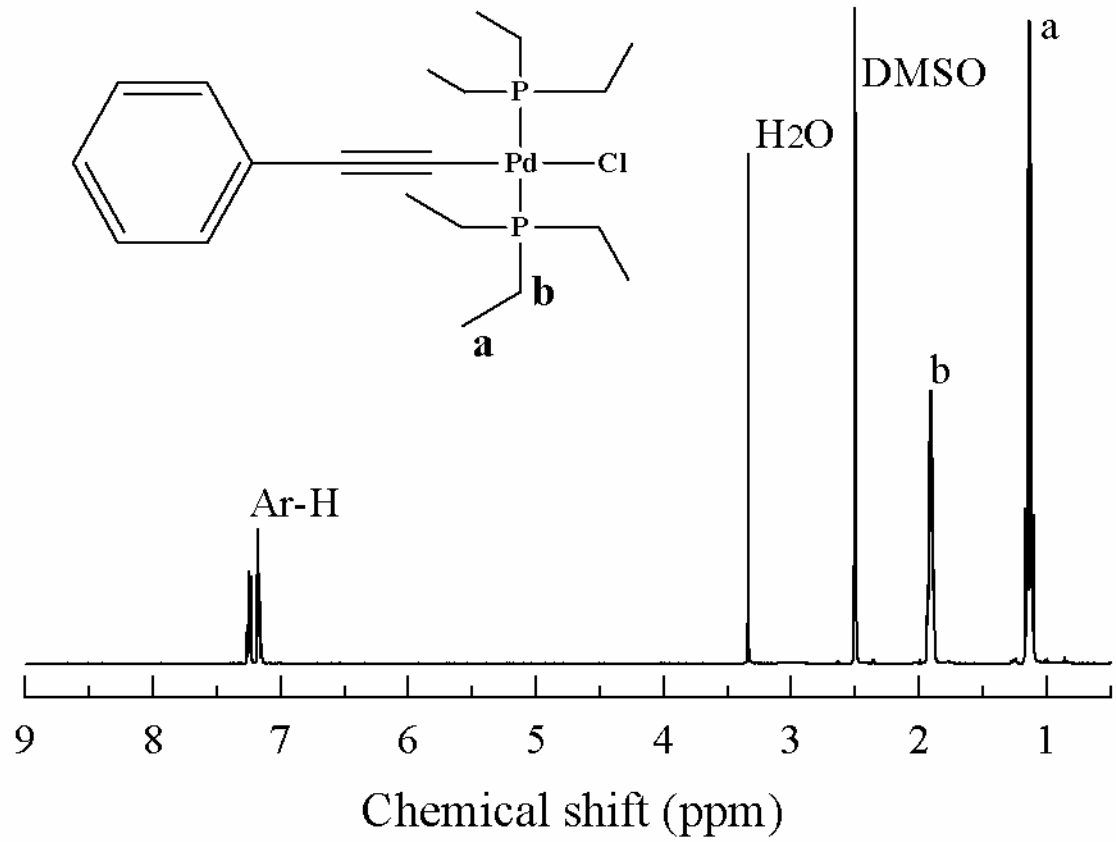

(b) ^{1}H NMR spectra of the intermediate **a**

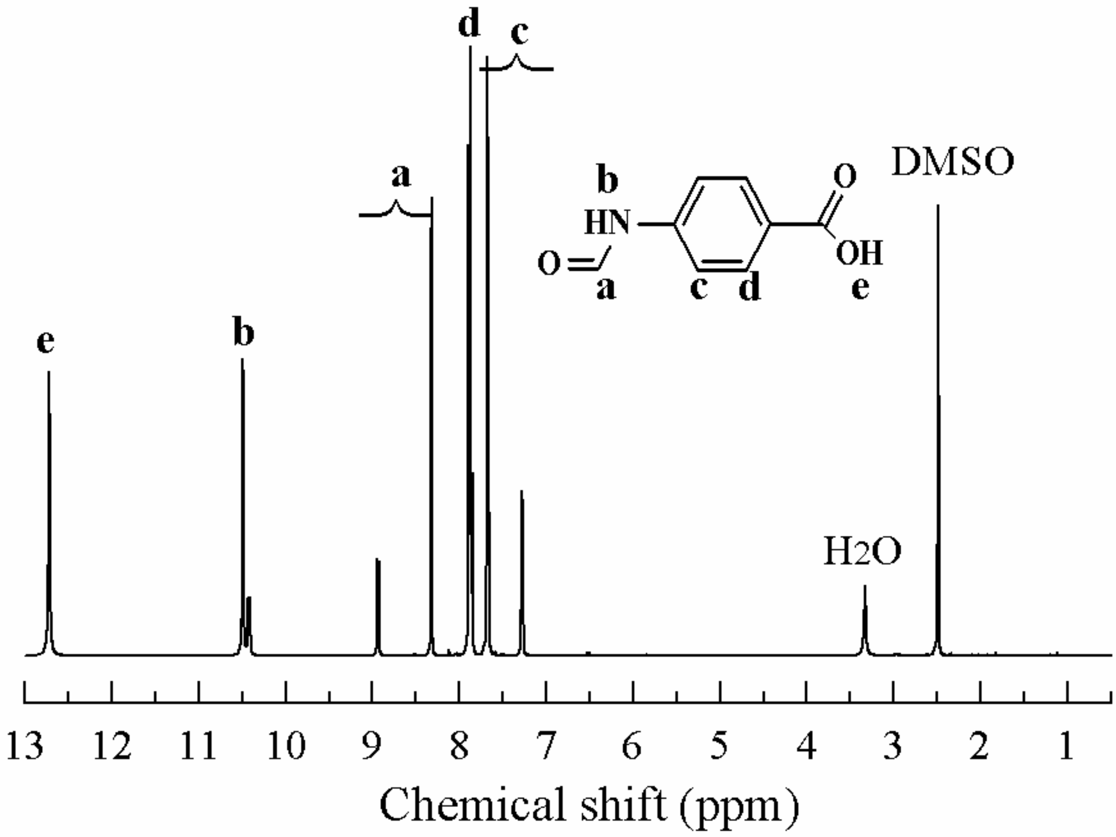

(c) ^{1}H NMR spectra of the intermediate **b**

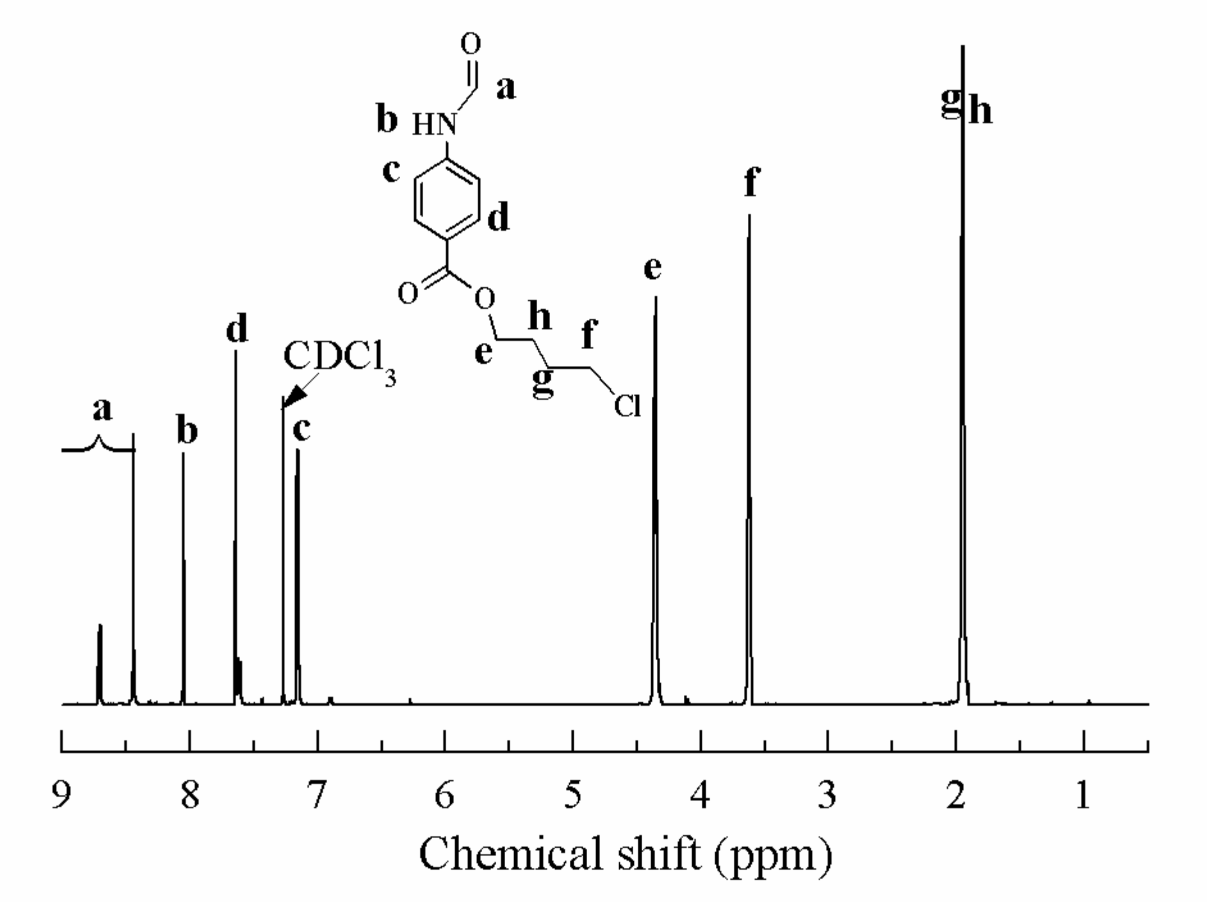

(d) ^{1}H NMR spectra of the isonitrile monomer

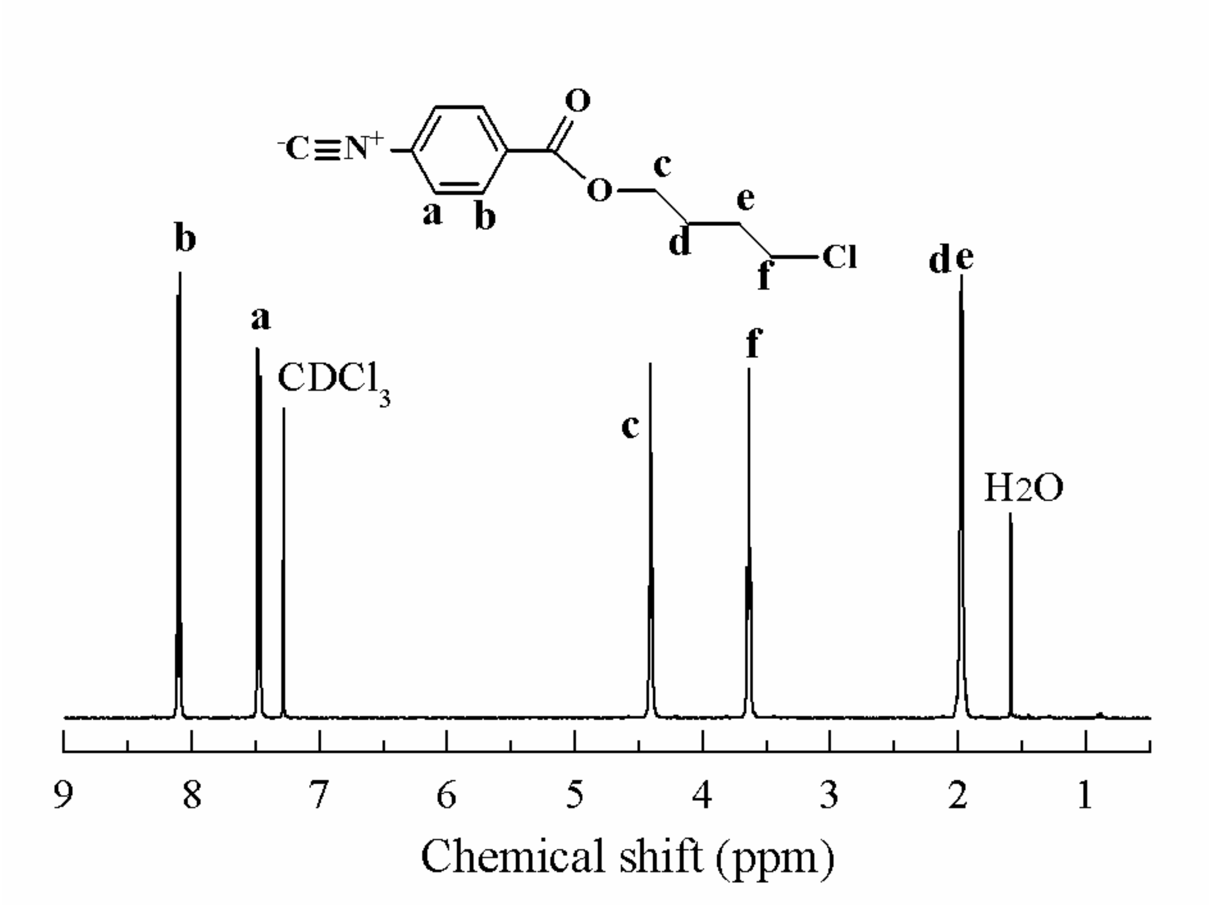

(e) ^{1}H NMR spectra of the L-P-M50

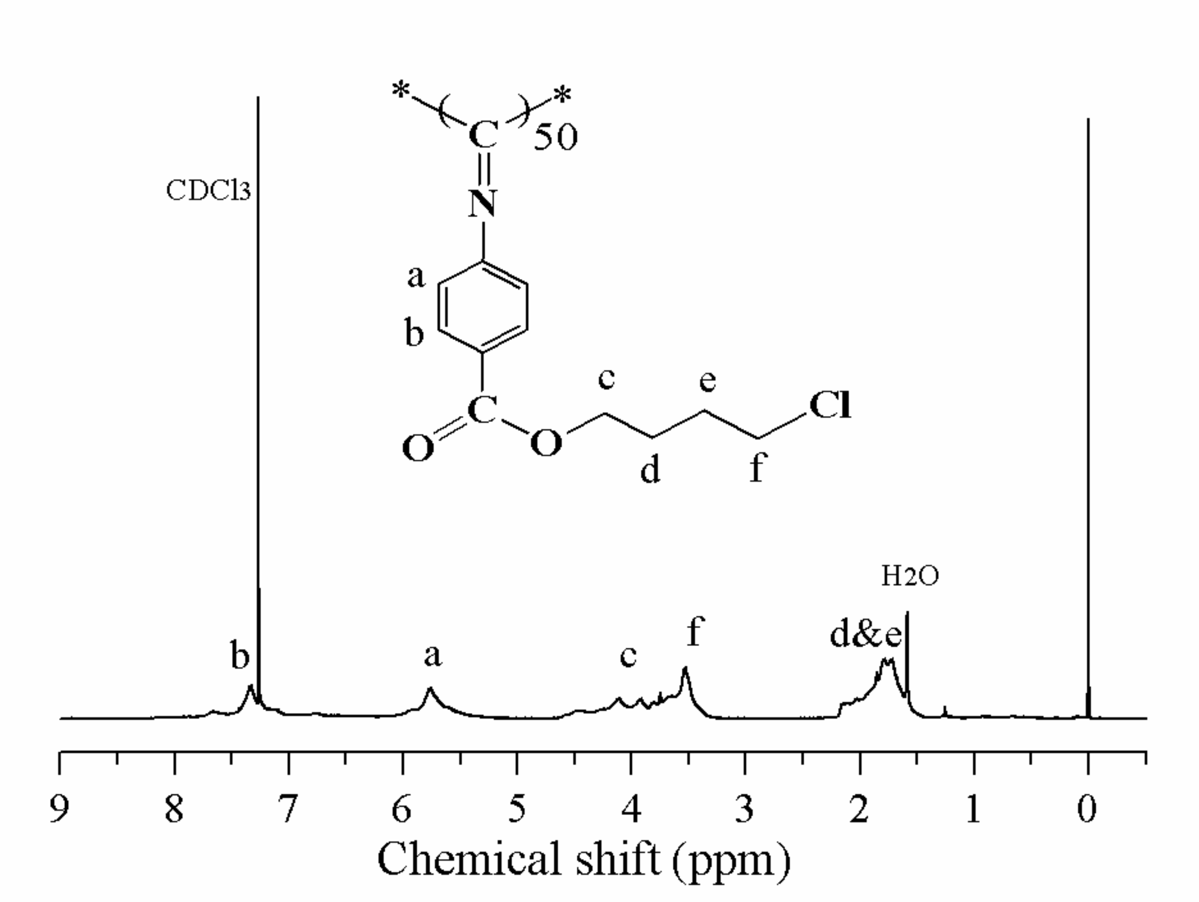

(f) ^{1}H NMR spectra of the L-PBnQAS-M$_{50}$

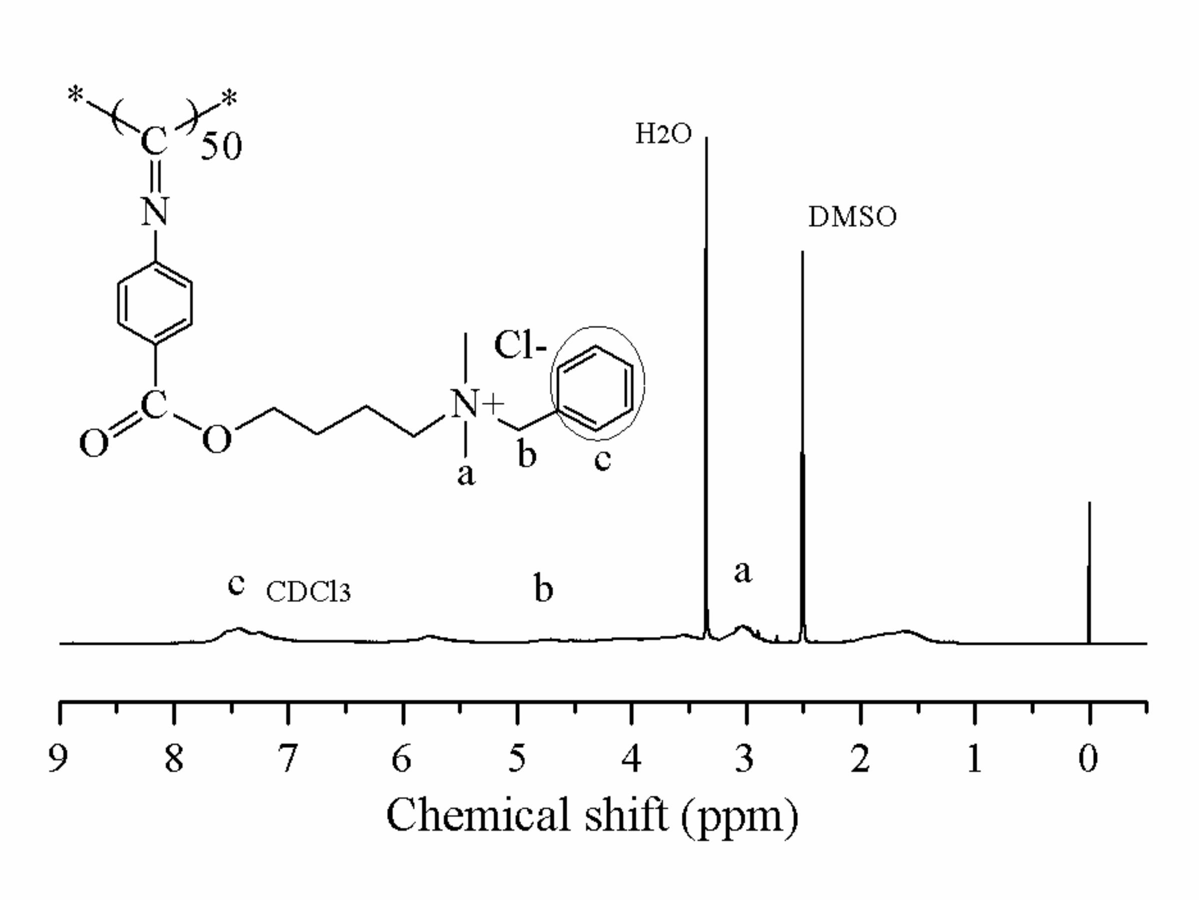

(g) ^{1}H NMR spectra of the L-PBuQAS-M50

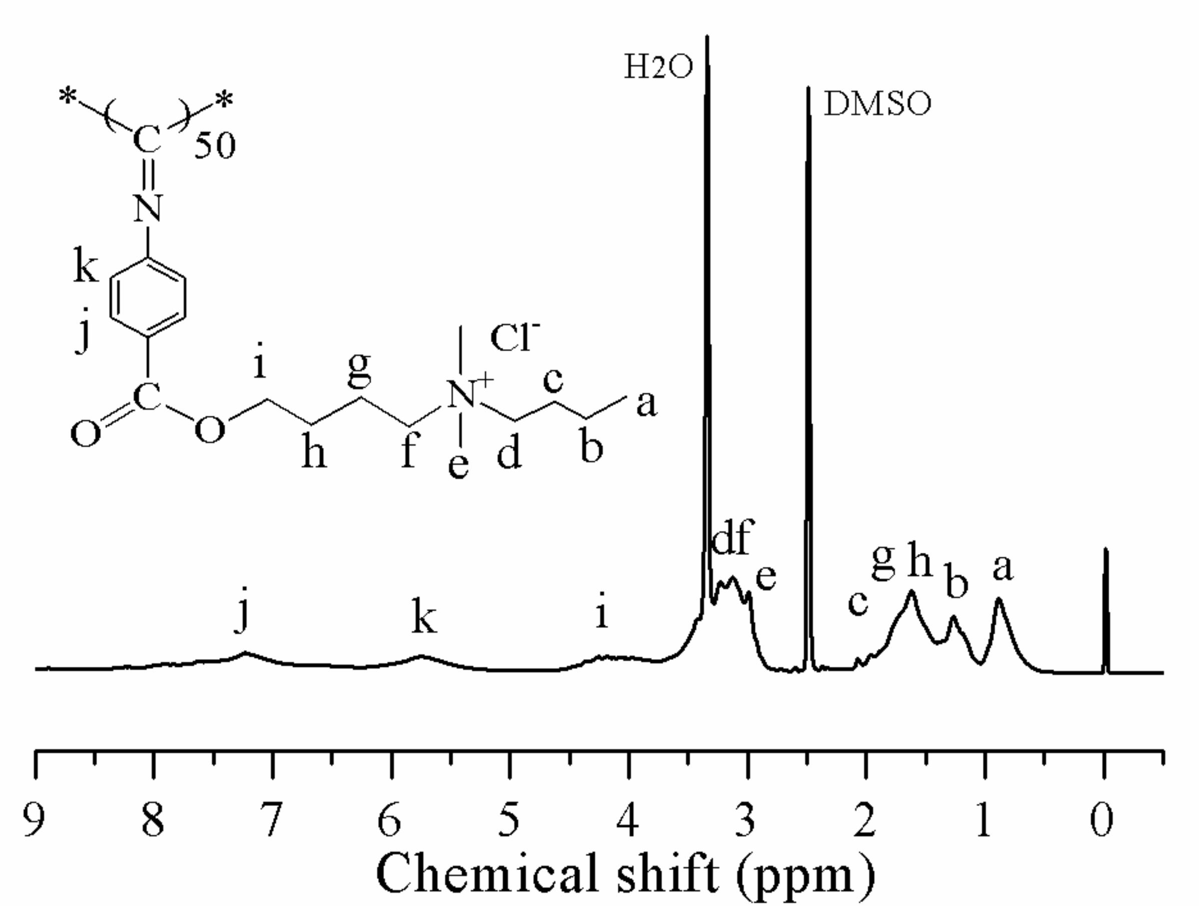

(h) ^{1}H NMR spectra of the L-POcQAS-M_{50}

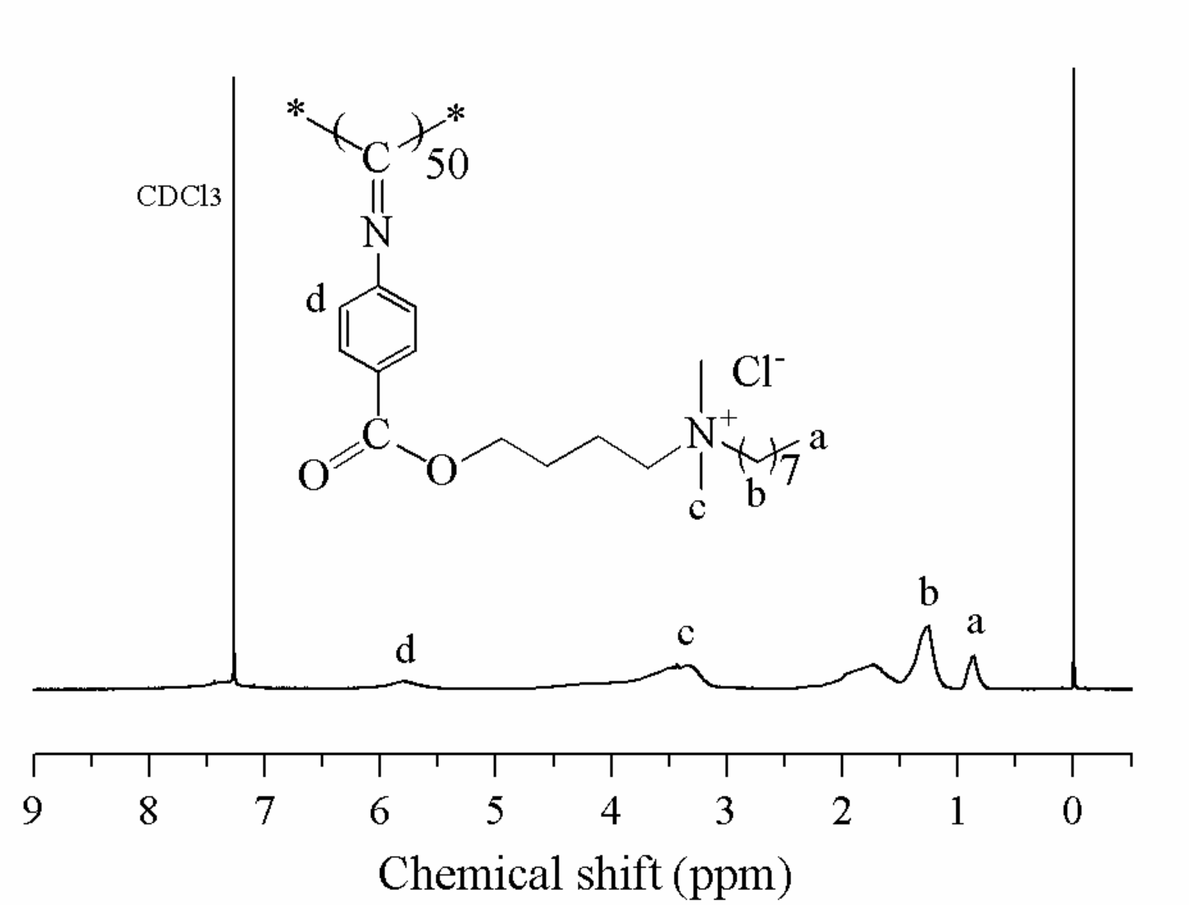

(i) ^{1}H NMR spectra of the L-PDBQAS-M$_{50}$

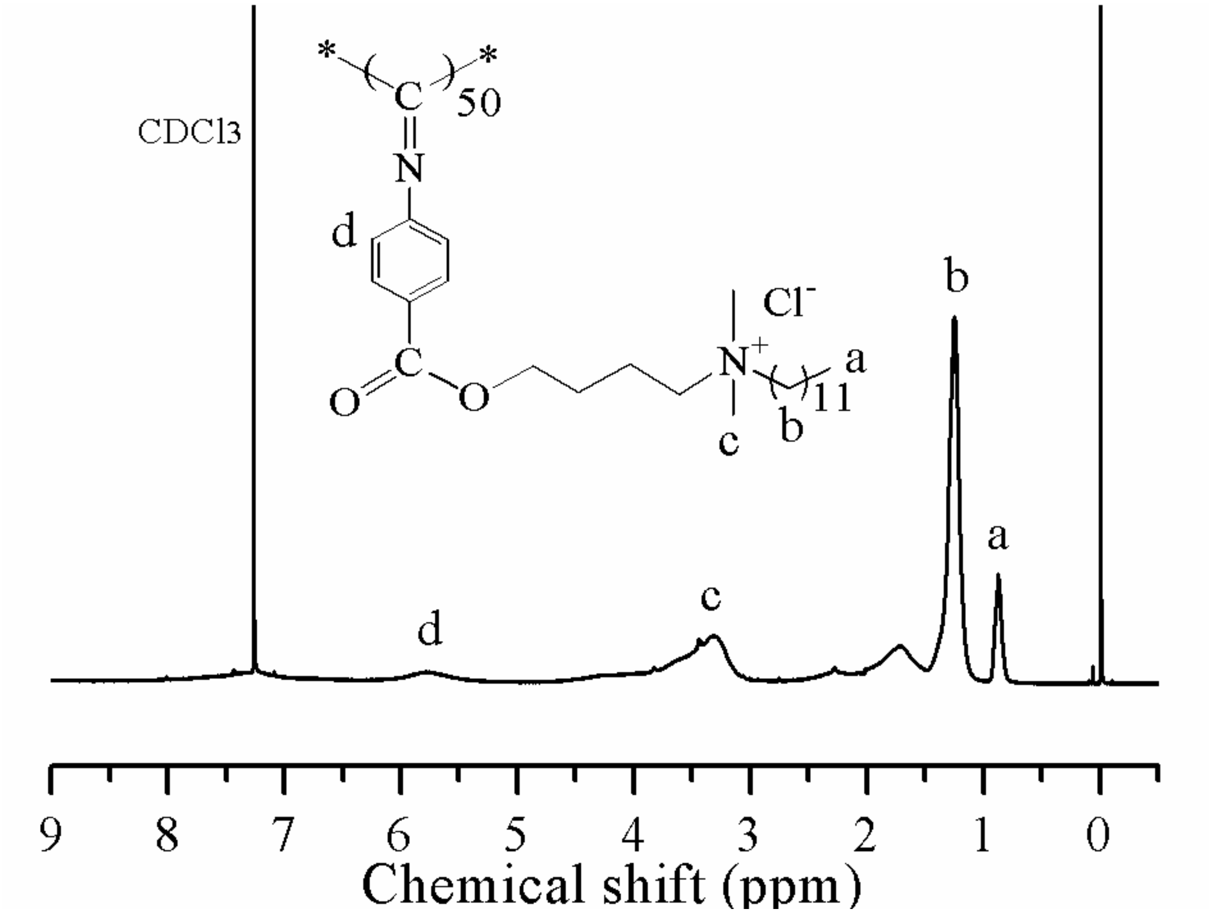

Figure S1. ^{1}H NMR spectra of all prepared compounds.
